# Occurrence & antibiogram of *Salmonella* Typhi & *S.* Paratyphi A isolated from Rourkela, Orissa

**Published:** 2011-04

**Authors:** S.S. Bhattacharya, Usha Das, B.K. Choudhury

**Affiliations:** Department of Microbiology, Ispat General Hospital, Rourkela, India; * *Dhenkanal (Auto) College,Dhenkanal, Orissa, India*

**Keywords:** Antibiogram, Multidrug resistance, *Salmonella* Paratyphi, *Solmonella* Typhi

## Abstract

**Background & objectives:**

Almost round-the-year occurrence of *Salmonella* Typhi and *Salmonella* Paratyphi A has been noticed in Rourkela since last 13 and five years respectively. The incidence of infection along with the antibiogram of these two serotypes in this area were carried out.

**Methods:**

The study was carried out at Ispat General Hospital, Rourkela, India, between January 2005 and December 2008 with 5340 blood samples collected from patients with suspected enteric fever and pyrexia of unknown origin. Isolation, identification and antibiogram of the causative organisms were performed according to standard bacteriological procedures.

**Results:**

A total of 298 *Salmonella* isolates showed an overall per cent positivity of 5.58. Multidrug resistance was found in 11.96 per cent and 15.62 per cent isolates of S. Typhi and S. Paratyphi A respectively. Less than 2 per cent isolates of *Salmonella* showed resistance to ciprofloxacin. A resistance of 3.0 to 6.25 per cent against third generation cephalosporins was observed among the salmonella isolates.

**Interpretation & conclusion:**

A round-the-year occurrence of *Salmonella* spp. in Rourkela might have been due to the presence of a considerable number of carriers in the locality, poor sanitation in nearby slum areas, and inadequate and contaminated community water supply at times. Higher degree of susceptibility among S. Typhi isolates against various antibiotics was encouraging, but increasing trend of resistance observed among S. Paratyphi A isolates was a matter of concern.

Enteric fever is prevalent world over and continues to be a major public health problem in developing countries. In India, though *Salmonella enterica* serotype Typhi remains the predominant *Salmonella* species causing enteric fever^1^,^2^, isolation of *Salmonella* enterica serotype Paratyphi A causing the same disease, has also been reported increasingly^3^,^4^.

In this retrospective study, almost round-the-year occurrence of *S.* Typhi and *S.* Paratyphi A and subsequently their antibiogram were studied for a period of four years (January 2005 to December 2008). There is no recent documentation regarding the occurrence of both *S.* Typhi and *S.* Paratyphi A infection from this part of western Orissa.

## Material and Methods

Between January 2005 and December 2008, a total of 5340 patients attending Out Patient Departments (OPDs) and wards of Paediatric and Medicine departments of Ispat General Hospital, Rourkela, provisionally diagnosed as having enteric fever or pyrexia of unknown origin (PUO) were included in this study.

*Samples*: A total of 5340 venous blood samples were included. Irrespective of a repeat sample, only one sample from each patient was included in the study. Since our objective was to find out the incidence of S. Typhi and S. Paratyphi A infection in this locality, only positive isolation was considered for the patients having both positive and negative samples.

Blood samples were collected in brain heart infusion broth with sterile precautions and incubated aerobically at 37°C for 48 h. Subcultures were done on blood agar, MacConkey agar and Salmonella-Shigella agar and incubated aerobically at 37°C for 18 to 24 h. In negative cases, subcultures were done for one week.

*S.* Typhi and *S.* Paratyphi A were isolated by conventional method as given in the WHO manual^5^. The isolates were identified by standard biochemical reactions and were confirmed by serotyping with factor sera^6^. Antibiotic susceptibility testing was carried out by Kirby-Bauer’s disk diffusion method^7^ with the modifications recommended by the Clinical Laboratory Standards Institute (CLSI)^8^. Antibiotics used in this study were ampicillin (A), chloramphenicol (C), co-trimoxazole (Co), gentamicin (G), amikacin (Ak), ciprofloxacin (Cf), cephotaxime (Ce) and ceftriaxone (Ci), supplied by Hi-Media Laboratories, Mumbai.

A standard strain of *Escherichia* coli (ATCC 25922) was used as quality control and included with each batch of tests.

A data bank was created in departmental computer. Month-wise isolation data of *S.* Typhi and *S.* Paratyphi A were studied and entered into the data bank every month. Comparative isolation data of these two organisms for each year were collected from the data bank. Information regarding age and sex of the patients, and provisional diagnosis of the disease was also made available in the data bank.

## Results and Discussion

Of the total patients studied, 3430 (64.23%) were children and 1910 (35.76%) were adults. The patients were diagnosed provisionally as having enteric fever (228, 76.5%), PUO (56, 18.8%) and relapse enteric fever (14, 4.7%) due to treatment failure. The incidence of enteric fever was higher in children with 182 cases (61%) compared to adults (116, 39%), while sex-wise distribution was not much different.

A total of 298 isolates of S. Typhi (234) and S. Paratyphi A (64) were obtained by blood culture from suspected cases of enteric fever and PUO, giving an overall per cent positivity of 5.58. Almost 74 per cent of isolates were from paediatric population (51.72% boys and 48.28% girls). Among adults, 52.57 per cent were male whereas 47.43 per cent were female.

Salmonellae were isolated almost throughout the year. The number of Isolation of both *S.* Typhi and *S.* Paratyphi A was highest in 2005 (9.2%) (Table I). Highest number of *S.* Paratyphi A (24) was isolated in 2005 and lowest (8) in 2007. In 2006, there was no isolation of *Salmonella* in November and December; and in 2007, none was isolated in December. Every year, most of the isolation of salmonella occurred in between March and July. In 2005, a surge in isolation was noticed also from August to December with a peak in November.

**Table 1:** Year-wise isolation of *S.* Typhi and *S.* Paratyphi A in Rourkela

Year	No. of blood samples	*S.* Typhi (%)	*S.* Paratyphi A(%)
2005	1502	114 (7.6)	24 (1.6)
2006	1232	53 (4.3)	17 (1.38)
2007	1180	34 (2.88)	8 (0.68)
2008	1426	33 (2.31)	15 (1.05)
Total	5340	234(4.38)	64 (1.2)

Multidrug resistance (MDR) was found in 12.75 per cent of salmonella isolates. Resistance to chloramphenicol was found in 8.97 and 23.44 per cent of *S.* Typhi and *S.* Paratyphi A isolates respectively. Resistance to ciprofloxacin was found to be less than 2 per cent in cases of both *S.* Typhi and *S.* Paratyphi A (Table II).

**Table 2:** Resistance pattern of *S.* Typhi and *S.* Paratyphi A isolates in Rourkela

Antibiotics	*S.* Typhi (%) (n=234)	*S.* Paratyphi A (%) (n=64)
Ampicilin	50 (21.36)	18 (28.12)
Co-trimoxazole	64 (27.35)	23 (35.94)
Chloramphenicol	21 (8.97)	15 (23.44)
Gentamicin	4 (1.71)	2 (3.12)
Amikacin	3 (1.28)	1 (1.56)
Ciprofloxacin	4 (1.71)	1 (1.56)
Cephotaxime	7 (3.0)	4 (6.25)
Ceftriaxone	7 (3.0)	3 (4.69)

In the present study, the number of isolation was generally more from March to July except in 2005, when a steady isolation of *Salmonella* spp. continued up to the end of December. The number of isolation of salmonella fell down from late August onwards and again started rising slowly from middle of January. This pattern was slightly different from our earlier report^9^. Poor sanitation in the nearby slum areas of this steel township and the presence of a large number of carriers (specially the food handlers) could be two important factors determining the occurrence of enteric fever - a finding comparable to the study from Namakkal, Tamil Nadu^10^. Another factor influencing this occurrence might be scanty and contaminated community water supply in summer and rainy season respectively.

Multidrug resistance (12.75%) seen in this study was comparable to our earlier reports^11^,^12^ and also to a recent report from Shimla^13^. In this study, the level of resistance to antimicrobials tested was similar in *S.* Typhi and *S.* Paratyphi A isolates except in case of chloramphenicol (*P*<0.05).

The susceptibility pattern among *S.* Typhi isolates to ampicillin and co-trimoxazole was similar to that reported in Nagpur^14^, whereas chloramphenicol sensitivity was comparatively higher in our study. The level of susceptibility among S.Paratyphi A isolates to ampicillin and chloramphenicol was comparable to that reported by Tankhiwale *et al*^15^ and that of ceftriaxone among *S.* typhi was comparable to that reported at Bangladesh^16^. A high degree (> 98%) of susceptibility to ciprofloxacin among both *S.* Typhi and *S.* Paratyphi A isolates was encouraging. Though a high susceptibility to aminoglycosides was shown by the salmonella isolates, a resistance of 3.0 to 6.25 per cent to third generation cephalosporins was a matter of concern.
